# Desigualdade de gênero: implicações na relação da insegurança alimentar e obesidade na cidade do Rio de Janeiro, Brasil

**DOI:** 10.1590/0102-311XPT131525

**Published:** 2026-07-06

**Authors:** Luana Teixeira Ghiggino, Talita Barbosa Domingos, Rosana Salles-Costa, Aline Alves Ferreira

**Affiliations:** 1 Universidade Federal do Rio de Janeiro, Rio de Janeiro, Brasil.

**Keywords:** Obesidade, Insegurança Alimentar, Fome, Gênero, Obesity, Food Insecurity, Hunger, Gender, Obesidad, Inseguridad Alimentaria, Hambre, Género

## Abstract

Este estudo buscou avaliar a associação da insegurança alimentar e obesidade em chefes de família adultos, considerando as diferenças entre homens e mulheres. Caracteriza-se como estudo transversal com dados do *I Inquérito sobre a Insegurança Alimentar no Município do Rio de Janeiro*. Foram analisados os indivíduos adultos (20-59 anos) responsáveis pelos domicílios (n = 1.026). A *Escala Brasileira de Insegurança Alimentar* foi utilizada para avaliar a segurança alimentar e os níveis de insegurança alimentar (leve e moderada/grave). As medidas de peso e estatura autorreferidas foram utilizadas para estimar a obesidade avaliada pelo índice de massa corporal. As associações entre os níveis de insegurança alimentar das famílias e a obesidade foram estimadas por modelos logísticos multinomiais (razão de risco relativo - RRR - e respectivos intervalos de confiança - IC95%), utilizando-se o software Stata. As mulheres com insegurança alimentar leve (RRR = 2,8; IC95%: 1,3-5,8) e insegurança alimentar moderada/grave (RRR = 2,5; IC95%: 1,0-6,4) tiveram maior risco de obesidade. A insegurança alimentar foi um fator de risco para obesidade somente entre as mulheres, sem resultado estatisticamente significativo entre os homens, evidenciando como a desigualdade de gênero amplia os efeitos adversos da insegurança alimentar das famílias chefiadas por mulheres, com comprometimento da qualidade e quantidade da sua alimentação.

## Abstract (en)

This study aimed to evaluate the association between food insecurity and obesity in adult heads of household considering the differences between men and women. This cross-sectional study used data from the *I Survey on Food Insecurity in the City of Rio de Janeiro*. Adults (aged 20-59 years) who headed households (n = 1,026) were analyzed. Food security and food insecurity levels (mild and moderate/severe) were assessed by the *Brazilian Food Insecurity Scale*. Self-reported weight and height were used to estimate obesity, assessed by body mass index. Associations between families’ food insecurity levels and obesity were estimated by multinomial logistic models (relative risk ratios - RRR - and their 95% confidence intervals - 95%CI) on Stata software. Women with mild (RRR = 2.8; 95%CI: 1.3-5.8) and moderate/severe food insecurity (RRR = 2.5; 95%CI: 1.0-6.4) had a higher risk of obesity. food insecurity constituted a risk factor for obesity only in women, with no statistically significant results in men, showing how gender inequality amplifies the adverse effects of food insecurity on female-headed households, compromising the quality and quantity of their diet.

## Abstract (es)

El objetivo de este estudio fue evaluar la asociación de la inseguridad alimentaria (IA) y la obesidad en adultos jefes de hogar en cuanto a diferencias entre hombres y mujeres. Estudio transversal con datos de la *I Encuesta de Inseguridad Alimentaria en el Municipio de Río de Janeiro*. Se analizaron a individuos adultos (20-59 años) responsables de hogares (n = 1.026). Se utilizó la *Escala Brasileña de Inseguridad Alimentaria* para evaluar la seguridad alimentaria y los niveles de inseguridad alimentaria (leve y moderada/grave). Las mediciones de peso y altura autoinformadas se utilizaron para estimar la obesidad evaluada por el índice de masa corporal. Las asociaciones entre los niveles de inseguridad alimentaria de las familias y la obesidad se estimaron mediante modelos logísticos multinomiales (razón de riesgo relativo -RRR- y respectivos intervalos de 95% de confianza -IC95%), utilizando el software Stata. Las mujeres con inseguridad alimentaria leve (RRR = 2,8; IC95%: 1,3-5,8) e inseguridad alimentaria moderada/grave (RRR = 2,5; IC95%: 1,0-6,4) tuvieron un mayor riesgo de obesidad. La inseguridad alimentaria fue un factor de riesgo para la obesidad solo entre las mujeres, sin resultados estadísticamente significativos entre los hombres, lo que muestra cómo la desigualdad de género aumenta los efectos adversos de la inseguridad alimentaria en las familias encabezadas por mujeres y compromete la calidad y cantidad de su alimentación.

## Introdução

Em 2023, 21,6 milhões de brasileiros enfrentavam algum grau de insegurança alimentar, e 3,2 milhões estavam em situação de fome ^1^. Embora esses números representem uma melhora do cenário da segurança alimentar em relação a anos anteriores ^2^, a insegurança alimentar ainda incide de forma desproporcional nos domicílios chefiados por mulheres ^1^. Essa maior vulnerabilidade feminina pode ser atribuída a fatores estruturais, como o acesso a recursos produtivos e a sobrecarga das responsabilidades domésticas e de cuidado familiar ^2^ ^,^ ^3^.

Na Região Sudeste, embora se observem indicadores socioeconômicos mais favoráveis em comparação a outras regiões do país, persistem profundas desigualdades sociais internas, sobretudo nos grandes centros urbanos, como o Município do Rio de Janeiro ^1^ ^,^ ^4^ ^,^ ^5^. Corroborando este cenário, em 2024, a proporção de famílias vivenciando a fome no Município do Rio de Janeiro superou as estimativas nacional e do próprio estado, evidenciando uma realidade local ainda mais crítica, especialmente nas famílias chefiadas por mulheres ^4^.

Paralelamente ao avanço da insegurança alimentar, observa-se o crescimento das taxas de obesidade no país. Dados do *Sistema de Vigilância de Fatores de Risco e Proteção para Doenças Crônicas por Inquérito Telefônico* (Vigitel) indicaram que 24,3% da população adulta brasileira apresentava obesidade, sendo mais prevalente entre as mulheres (24,8%) ^6^. No Estado do Rio de Janeiro, os indicadores foram ainda mais elevados, alcançando 26,2% da população, com 27% entre mulheres e 25,2% entre homens ^6^. Esses números confirmam não apenas a tendência nacional de aumento da obesidade, mas também revelam desigualdades de gênero e territoriais que dificultam o enfrentamento desse grave problema de saúde pública ^7^.

Embora historicamente associada à desnutrição, a insegurança alimentar também tem sido associada, de forma aparentemente contraditória, à obesidade em adultos ^8^ ^,^ ^9^. Essa correlação é observada em mulheres ^7^, sugerindo que fatores específicos de gênero, como diferenças no acesso à saúde, alimentação e condições de vida podem estar contribuindo para a vulnerabilidade feminina à obesidade em contextos de insegurança alimentar ^10^. A principal hipótese para essa associação é a da escassez de recursos, que leva ao consumo de alimentos ultraprocessados, mais acessíveis economicamente e com menor valor nutricional, contribuindo para o aumento da obesidade, sobretudo entre populações em situação de vulnerabilidade ^11^ ^,^ ^12^. As mulheres podem ser particularmente afetadas nesse contexto, uma vez que, historicamente, assumem maior responsabilidade pela alimentação familiar, frequentemente enfrentam desvantagens socioeconômicas, e tendem a vivenciar níveis mais severos de insegurança alimentar, aumentando seu risco de obesidade ^7^ ^,^ ^10^.

Apesar do reconhecimento de que somente as mulheres são desproporcionalmente afetadas pela relação entre insegurança alimentar e obesidade, poucos estudos investigam essa associação à luz das desigualdades de gênero, sobretudo em nível municipal. A análise nesse recorte territorial permite captar nuances locais das iniquidades em saúde, revelando como contextos socioeconômicos e culturais específicos moldam os padrões de vulnerabilidade. Ao mesmo tempo, oferece subsídios fundamentais para o delineamento de políticas públicas mais equitativas e territorializadas. Diante das elevadas prevalências de insegurança alimentar e obesidade observadas em todo o território nacional, torna-se fundamental aprofundar a compreensão sobre a interrelação entre esses dois problemas de saúde pública. Nesse contexto, este estudo buscou analisar a associação entre a insegurança alimentar e a obesidade, considerando as desigualdades entre homens e mulheres, na cidade do Rio de Janeiro.

## Métodos

### Desenho do estudo

Estudo transversal de base populacional executado com os dados do *I Inquérito sobre a Insegurança Alimentar no Município do Rio de Janeiro*, realizado entre novembro de 2023 e janeiro de 2024, que investigou uma amostra representativa de 2 mil domicílios do Município do Rio de Janeiro ^4^.

O Rio de Janeiro ocupa posição de destaque no cenário nacional, tanto do ponto de vista econômico quanto do histórico e sociocultural. Reconhecido mundialmente por suas belezas naturais, manifestações culturais e pelo turismo, também figura entre os municípios com maior produto interno bruto (PIB) do Brasil ^5^. No entanto, esse protagonismo econômico contrasta com profundas desigualdades sociais, sendo também um dos territórios mais desiguais do país ^13^. Para o cálculo amostral, levou-se em conta a distribuição dos setores censitários e considerando os bairros das cinco zonas de moradia da cidade ^4^.

O delineamento amostral considerou uma prevalência de 15,9% de insegurança alimentar grave no Estado do Rio de Janeiro, adotando intervalo de 95% de confiança (IC95%) e margem de erro máxima de 4,9 pontos percentuais por zona de moradia e de 2,2 pontos percentuais na amostra total. Detalhes sobre o desenho amostral e os procedimentos estão disponíveis no relatório final do estudo ^4^. Neste estudo, foram incluídos apenas os dados dos chefes de família adultos (20-59 anos), excluindo as gestantes identificadas (0,4%), resultando em uma amostra final de 1.026 indivíduos.

### Coleta de dados

As entrevistas foram feitas face a face por entrevistadores treinados, utilizando dispositivos móveis para registro e entrada de dados, com o morador responsável pelo domicílio ou aquele que sabia responder informações sobre a família. O banco de dados passou por controle de qualidade dos dados por pesquisador treinado e com experiência na função, identificando possíveis inconsistências das informações ^4^.

### Variáveis de estudo

A *Escala Brasileira de Insegurança Alimentar* (EBIA) foi utilizada para classificar a situação dos domicílios em segurança alimentar e níveis de insegurança alimentar (leve, moderada e grave) ^4^. A EBIA é composta por 14 perguntas dicotômicas, validada no país desde 2004 para uso em estudos populacionais ^13^. As perguntas foram feitas para a pessoa de referência na família que era responsável pela compra e preparação das refeições ^4^.

O peso e estatura dos chefes de família foram avaliados de forma autorreferida, sendo calculado o índice de massa corporal (IMC) (peso em kg/estatura em m^2^) para avaliar o estado nutricional antropométrico, com base nos pontos de corte da Organização Mundial da Saúde (OMS) ^14^ em: eutrófico (IMC entre 18,6-24,9kg/m^2^), sobrepeso (IMC entre 25-29,9kg/m^2^), e obesidade (IMC ≥ 30kg/m^2^). A categoria de baixo peso não foi incluída na análise estatística devido à baixa representatividade amostral (1,7%).

A variável sexo dos chefes de família foi analisada como “mulher/homem”, tal como disponibilizado nas perguntas do inquérito ^4^, seguindo a classificação adotada pelo Instituto Brasileiro de Geografia e Estatística (IBGE) ^1^. Embora o sexo seja considerado uma categoria descritiva e o gênero uma categoria analítica, optou-se por essa categorização para embasar a análise das designações “feminino” e “masculino”. A base teórica está ancorada em abordagens feministas que articulam as desigualdades de gênero com outros marcadores sociais, como raça e classe, compondo um sistema de opressões interligadas e simultâneas ^15^ ^,^ ^16^. Essas abordagens reconhecem, ainda, as relações de poder assimétricas entre homens e mulheres que sustentam dinâmicas de dominação e hierarquia social, extrapolando a lógica binária do sexo ^15^.

Outros fatores socioeconômicos e demográficos também foram avaliados, como: faixa etária em anos (20-29,9; 30-39,9; 40-59,9), raça/cor autorreferida segundo a definição do IBGE ^1^ (branca, preta e parda), anos de estudo (≤ 4, 5-8, 9-11, ≥ 12), recebimento do Bolsa Família, renda *per capita* familiar com base no salário mínimo vigente durante o estudo de R$ 1.320,00 (< 1/4, 1/4-1, 1-2 e ≥ 2), presença de menores de cinco anos, número de moradores no domicílio e zonas de moradia. No Município do Rio de Janeiro, o território é dividido por regiões administrativas caracterizadas como zonas de moradias (central, sul, norte, sudoeste e oeste). Algumas delas são caracterizadas por melhores perfis socioeconômicos e famosas no turismo internacional (zona sul), enquanto outras apresentam baixos indicadores sociais e econômicos (zonas norte e oeste). Determinadas zonas concentram bairros de caráter mais comercial, como a central e a norte, enquanto outras combinam áreas nobres, com desenvolvimento mais expressivo, como ocorre na zona sudoeste ^5^. As populações indígena e amarela foram excluídas da análise pela amostra não ser representativa para as categorias de raça/cor para o município (1,5%).

### Análise de dados

As análises foram realizadas no software Stata, versão 16.0 (https://www.stata.com), com o comando *Survey Data Analysis* (prefixo *svy*). Foram estimadas a prevalência e os IC95% para cada variável socioeconômica, utilizando o teste qui-quadrado (χ^2^) para comparar a relação entre os estratos de IMC. As covariáveis que apresentaram nível de significância p < 0,20 na análise inicial foram incluídas na análise multivariável ^17^. Posteriormente, foram utilizados modelos de regressão logística multinomial para verificar as variáveis associadas aos estratos de segurança alimentar e insegurança alimentar (leve e moderada/grave) e de IMC, considerando os modelos estratificados por homens e mulheres, além de ajustes para as covariáveis. O agrupamento da insegurança alimentar moderada/grave seguiu a metodologia utilizada por diferentes estudos na temática ^2^ ^,^ ^18^.

Os resultados foram expressos pela estimativa da razão de risco relativo (RRR) e seus respectivos IC95%, considerando valores significantes as associações com p < 0,05. Os modelos finais foram comparados por meio do critério de informação de Akaike (AIC) ^19^. Foram testadas as possíveis interações após a inclusão simultânea das variáveis idade, raça/cor da pele, escolaridade, recebimento de benefício do Bolsa Família, presença de crianças menores de cinco anos e zona de moradia.

### Aspectos éticos

Este estudo foi elaborado com base na *Resolução nº 510/2016* do Conselho Nacional de Saúde, art. 2º, inciso XXV, e aprovado pelo Comitê de Ética em Pesquisa do Hospital Universitário Clementino Fraga Filho da Universidade Federal do Rio de Janeiro, em maio de 2022 (CAEE: 54473421.6.0000.5257), sendo conduzido de acordo com todas as diretrizes éticas para estudos com seres humanos no país. Todos os participantes foram previamente informados sobre os objetivos, riscos e benefícios da pesquisa e assinaram o Termo de Consentimento Livre e Esclarecido (TCLE) antes da participação. Mais detalhes estão disponíveis no relatório final do estudo ^4^.

## Resultados

A população estudada era composta por 1.026 indivíduos. Observou-se que mais da metade dos chefes de família tinha entre 40 e 59 anos (54,3%), era do sexo masculino (51%), não recebia Bolsa Família (76,8%) e tinha de 1 a 2 moradores no domicílio (51%). A maioria se autodeclarou parda (45,6%), com 9-11 anos de estudo (37,8%) e recebia renda familiar *per capita* entre 1/4 e 1 salário mínimo (42,7%).

A análise do estado nutricional antropométrico segundo as características socioeconômicas, demográficas e de (in)segurança alimentar evidenciou que a proporção de indivíduos em eutrofia foi menor entre faixas etárias mais avançadas, enquanto as prevalências de sobrepeso e obesidade foram maiores. Verificou-se maior prevalência de obesidade quando o responsável pela família era autodeclarado preto (20%; IC95%: 16,3-24,4), com ≤ 4 anos de estudo (27,4%; IC95%: 17,6-40,1), ou beneficiário do Bolsa Família (25,6%; IC95%: 19,5-32,9). Além disso, as prevalências de sobrepeso (35,5%; IC95%: 26,6-45,6) e obesidade (29,1%; IC95%: 20,9-38,9) entre os adultos foram mais expressivas naqueles em insegurança alimentar moderada/grave, bem como nos residentes das zonas central (26,5%; IC95%: 20,9-33,0) e oeste (25,6%; IC95%: 20,3-31,7) do município (Tabela 1).

**Tabela 1:** Prevalências ponderadas do estado nutricional antropométrico (%) segundo características socioeconômicas, demográficas, segurança alimentar e níveis de insegurança alimentar. *I Inquérito sobre a Insegurança Alimentar no Município do Rio de Janeiro*, Rio de Janeiro, Brasil, 2024.

Variáveis	Eutrofia	Sobrepeso	Obesidade	Valor de p
	% (IC95%)	% (IC95%)	% (IC95%)	
Idade (anos)				< 0,05 *
20-29,9	58,4 (50,1-66,3)	26,9 (20,2-34,9)	14,7 (9,6-21,7)	
30-39,9	36,1 (30,2-42,5)	45,5 (39,2-51,9)	18,4 (13,9-24,0)	
40-59,9	36,6 (32,0-41,4)	40,4 (35,8-45,2)	23,0 (19,3-27,3)	
Sexo				0,506
Masculino	38,2 (33,5-43,1)	41,5 (36,8-46,4)	20,3 (16,6-24,5)	
Feminino	42,1 (37,2-47,1)	37,9 (33,2-42,9)	20,0 (16,3-24,4)	
Raça/Cor				< 0,05 *
Branca	43,7 (37,6-50,0)	41,0 (35,0-47,3)	15,2 (11,3-20,3)	
Preta	34,9 (28,3-42,2)	37,7 (30,9-45,0)	27,4 (21,3-34,4)	
Parda	40,2 (35,2-45,4)	40,1 (35,1-45,3)	19,7 (15,9-24,2)	
Escolaridade (anos)				0,057
≤ 4	29,5 (18,6-43,3)	43,1 (30,6-56,5)	27,4 (17,6-40,1)	
5-8	43,4 (36,8-50,2)	34,0 (27,9-40,6)	22,7 (17,6-28,7)	
9-11	36,7 (31,4-42,3)	44,7 (39,2-50,4)	18,5 (14,6-23,4)	
≥ 12	45,7 (39,4-52,2)	36,1 (30,2-42,5)	18,2 (13,6-24,0)	
Recebe Bolsa Família				< 0,05 *
Sim	42,6 (35,3-50,3)	31,8 (25,2-39,2)	25,6 (19,5-32,9)	
Não	39,6 (35,8-43,6)	41,5 (37,7-45,5)	18,9 (16,0-22,1)	
Renda familiar *per capita* (salários mínimos) **				0,589
< 1/4	38,3 (24,3-54,6)	33,1 (20,0-49,4)	28,6 (16,8-44,1)	
1/4-1	39,9 (34,4-45,7)	38,6 (33,2-44,4)	21,5 (17,2-26,5)	
1-2	44,2 (37,6-51,1)	37,5 (31,2-44,3)	18,2 (13,6-24,0)	
≥ 2	41,3 (34,3-48,7)	42,0 (35,0-49,4)	16,6 (11,9-22,7)	
Presença de crianças ≤ 5 anos				0,162
Sim	48,9 (39,5-58,3)	33,4 (25,1-43,0)	17,7 (11,6-26,0)	
Não	39,2 (35,6-42,9)	40,3 (36,7-44,0)	20,5 (17,7-23,7)	
Moradores no domicílio				0,415
1-2	42,9 (38,4-47,6)	37,5 (33,1-42,1)	19,6 (16,1-23,6)	
3-4	37,1 (31,8-42,7)	42,8 (37,3-48,5)	20,1 (16,0-24,9)	
≥ 5	40,1 (27,3-54,5)	34,3 (22,7-48,2)	25,6 (15,3-39,5)	
EBIA				< 0,05 *
Segurança alimentar	42,0 (38,0-46,2)	41,8 (37,8-46,0)	16,2 (13,4-19,4)	
Insegurança alimentar leve	38,4 (30,5-47,1)	33,0 (25,6-41,3)	28,6 (21,6-36,7)	
Insegurança alimentar moderada/grave	35,4 (26,6-45,4)	35,5 (26,6-45,6)	29,1 (20,9-38,9)	
Zonas de moradia				< 0,05 *
Central	44,1 (37,4-51,0)	29,4 (23,6-36,0)	26,5 (20,9-33,0)	
Sul	41,7 (35,1-48,7)	44,4 (37,7-51,3)	13,9 (9,8-19,4)	
Norte	42,6 (35,7-48,7)	39,6 (32,9-46,8)	17,9 (13,0-23,9)	
Sudoeste	35,9 (29,8-42,5)	44,8 (38,3-41,8)	19,3 (14,6-25,2)	
Oeste	39,0 (32,9-45,5)	35,4 (36,0-42,9)	25,6 (20,3-31,7)	

EBIA: *Escala Brasileira de Insegurança Alimentar*; IC95%: intervalo de 95% de confiança.

Nota: os valores de p se referem ao teste χ^2^ para diferenças percentuais; segurança alimentar e níveis de insegurança alimentar são classificadas de acordo com a EBIA ^13^.

* Valor de p < 0,05, o resultado é estatisticamente significativo;

** Salário mínimo vigente em novembro de 2023: R$ 1.320,00.

**Figura 1:**
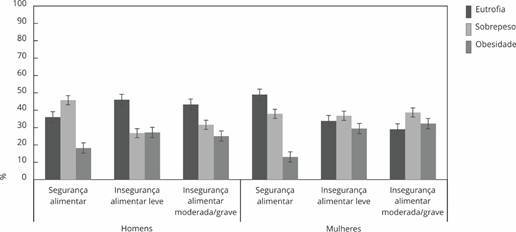
Estado nutricional antropométrico segundo os níveis de segurança e insegurança alimentar entre homens e mulheres. *I Inquérito sobre a Insegurança Alimentar no Município do Rio de Janeiro*, Rio de Janeiro, Brasil, 2024.

Com base na Figura 1, que apresenta a prevalência das categorias de IMC segundo os estratos de segurança alimentar e níveis de insegurança alimentar, estratificado por mulheres e homens, observou-se que as prevalências de sobrepeso (38,7%; IC95%: 26,6-52,5) e obesidade (32,3%; IC95%: 21,3-45,8) foram significativamente maiores (p < 0,05) entre mulheres com insegurança alimentar moderada/grave. Entre homens a relação não foi estaticamente significativa (p = 0,007).

As associações entre a relação da insegurança alimentar e excesso de peso se mantiveram após os ajustes por idade e fatores sociodemográficos (Tabela 2). Entre as mulheres, a RRR de apresentar obesidade foi 2,76 vezes maior naquelas em insegurança alimentar leve e de 2,57 vezes maior naquelas em insegurança alimentar moderada/grave. Em relação ao sobrepeso, a RRR foi 1,76 vez maior entre as mulheres com insegurança alimentar moderada/grave, demonstrando uma maior vulnerabilidade entre as mulheres.

Fonte: elaboração própria. Nota: segurança alimentar e níveis de insegurança alimentar são classificadas de acordo com a *Escala Brasileira de Insegurança Alimentar* (EBIA) ^13^.

**Tabela 2:** Estimativa dos valores de razão de risco relativo (RRR) e seus respectivos intervalos de 95% de confiança (IC95%) da relação entre as proporções de sobrepeso e obesidade, com relação à eutrofia, segundo os níveis de segurança alimentar e insegurança alimentar, entre mulheres e homens. *I Inquérito sobre a Insegurança Alimentar no Município do Rio de Janeiro*, Rio de Janeiro, Brasil, 2024.

Variáveis	Mulheres				Homens			
	RRR (IC95%)	Valor de p	RRRa (IC95%)	Valor de p	RRR (IC95%)	Valor de p	RRRa (IC95%)	Valor de p
Sobrespeso								
Insegurança alimentar leve	1,41 (0,78-2,53)	0,251	1,47 (0,78-2,80)	0,235	0,46 (0,23-0,93)	< 0,05 *	0,55 (0,26-1,18)	0,127
Insegurança alimentar moderada/grave	1,73 (0,85-3,50)	0,129	1,71 (0,76-3,88)	0,196	0,57 (0,27-1,21)	0,144	0,77 (0,33-1,76)	0,531
Obesidade								
Insegurança alimentar leve	1,41 (0,78-2,53)	0,251	2,76 (1,32-5,77)	< 0,05 *	1,16 (0,55-2,48)	0,693	1,76 (0,78-3,96)	0,173
Insegurança alimentar moderada/grave	1,73 (0,85-3,50)	0,129	2,57 (1,02-6,43)	< 0,05 *	1,14 (0,49-2,64)	0,754	1,96 90,73-5,30)	0,183

RRRa: razão de risco relativo ajustado (modelos ajustados para idade, raça/cor, escolaridade, recebimento de Bolsa Família, presença de crianças menores de cinco anos e zonas de moradia).

Nota: segurança alimentar e níveis de insegurança alimentar são classificadas de acordo com a *Escala Brasileira de Insegurança Alimentar* (EBIA) ^13^.

* Valor de p < 0,05, o resultado é estatisticamente significativo.

## Discussão

Os resultados deste estudo indicaram que a presença de insegurança alimentar nas famílias está associada à obesidade entre as mulheres, mas não entre os homens. Esses achados reforçam estudos anteriores que identificam as famílias chefiadas por mulheres como um dos grupos sociais mais vulnerabilizados na população brasileira e, portanto, mais suscetíveis às múltiplas expressões da insegurança alimentar ^3^.

A relevância deste trabalho reside na análise específica do contexto urbano do Município do Rio de Janeiro, fornecendo dados locais que aprofundam a compreensão das vulnerabilidades das mulheres e sustentam o planejamento de ações e políticas públicas direcionadas à prevenção da obesidade e à promoção da segurança alimentar.

Essa condição de maior vulnerabilidade pode ser compreendida à luz do conceito de divisão sexual do trabalho ^15^. Mesmo quando inseridas no mercado de trabalho formal, as mulheres tendem a ocupar posições de menor remuneração e continuam sendo as principais responsáveis pelo cuidado e pela alimentação da família ^15^ ^,^ ^16^. A tripla carga de trabalho impõe restrições significativas de tempo, dificulta o acesso a serviços públicos de socialização do cuidado e intensifica a exposição a condições socioeconômicas precárias, contribuindo para a perpetuação da pobreza entre esse grupo ^3^ ^,^ ^20^.

Historicamente, a responsabilidade pela provisão econômica do domicílio foi culturalmente associada aos homens, que eram aqueles que trabalhavam ^21^. Os processos de industrialização, períodos de guerra, controle da natalidade e mobilização feminista, principalmente na década de 1960, fizeram as mulheres ingressarem no mercado de econômico, passando a complementar a renda familiar, assumindo o papel de coprovedoras no lar. Entretanto, o que estava associado à emancipação ou autonomia feminina sobrecarrega as mulheres no que se refere aos aspectos físico e emocional ^16^ ^,^ ^17^.

Com a mudança na composição familiar, sobretudo pelo abandono e ausência de responsabilidade dos homens quando há filhos, a renda das mulheres passou a ser a principal fonte de sustento de seus lares, ao mesmo tempo em que a chefia feminina corresponde a 50,8% dos domicílios brasileiros ^1^ ^,^ ^22^. Esse cenário corrobora para inúmeras condições de desigualdades sociais observadas entre famílias chefiadas por mulheres e homens, incluindo a insegurança alimentar ^21^ ^,^ ^23^. Quando assumem a posição de chefes de família, as mulheres enfrentam maiores dificuldades no acesso a recursos financeiros e possuem a responsabilidade social pelo cuidado familiar, aprofundando a menor participação social, menor representação política e contribuindo para a desigualdade de gênero ^16^ ^,^ ^22^ ^,^ ^23^.

Neste estudo, apenas as mulheres apresentaram maior prevalência de sobrepeso e obesidade independentemente do nível de insegurança alimentar. Esse padrão já foi identificado em estudos anteriores, principalmente nos Estados Unidos, que apontam maior propensão à obesidade em mulheres em insegurança alimentar, quando comparadas com aquelas em segurança alimentar, não sendo observada associação significativa entre os homens ^7^ ^,^ ^9^ ^,^ ^10^.

Drewnowski & Darmon ^24^ apontam que mulheres de famílias em situação de pobreza tendem a recorrer com maior frequência a alimentos ultraprocessados, por serem amplamente disponíveis e mais acessíveis financeiramente. Em contextos de insegurança alimentar, é comum que as mulheres priorizem a alimentação dos filhos em detrimento da própria, o que pode aumentar sua suscetibilidade à obesidade nas fases de retomada do acesso regular aos alimentos ^24^ ^,^ ^25^ ^,^ ^26^. Dessa forma, o gênero emerge como um determinante central na relação entre insegurança alimentar e obesidade, evidenciando como as mulheres, mais expostas a essas condições, enfrentam maiores riscos.

Outro achado relevante deste estudo foi a maior prevalência de sobrepeso e obesidade entre os adultos responsáveis pelo domicílio em insegurança alimentar moderada/grave residentes nas zonas central e oeste da cidade. As zonas de moradia são concebidas como unidades territoriais essenciais para a organização do espaço urbano, desempenham um papel central na compreensão dos determinantes sociais da saúde, contribuindo para a análise dos padrões alimentares e da prevalência de doenças associadas ao excesso de peso no município ^5^ ^,^ ^27^.

Embora a zona central apresente melhor acesso a serviços e infraestrutura, os processos de intensa urbanização, segregação socioespacial e expulsão de populações de baixa renda para zonas periféricas comprometeram a qualidade de vida em diversas localidades. Esses processos, aliados à dificuldade de acesso a alimentos saudáveis e à escassez de espaços adequados para a prática de atividades físicas, podem ter favorecido comportamentos alimentares inadequados e o aumento do sedentarismo. Já a zona oeste caracteriza-se por um histórico de urbanização tardia e desordenada, piores indicadores socioeconômicos e infraestrutura precária, além de registrar uma das maiores proporções de fome no município (16,3%) ^13^. Essas desigualdades territoriais contribuem para padrões alimentares inadequados e maior risco de obesidade em contextos de insegurança alimentar ^28^.

Os resultados também reforçam a necessidade de políticas públicas que não se restrinjam apenas à redução da fome na população, mas que também contemplem, de forma integrada, as vulnerabilidades específicas que contribuem para o aumento da obesidade entre mulheres em áreas urbanas, como o Município do Rio de Janeiro avaliado neste estudo. Nesse contexto, combinar o enfrentamento da obesidade com ações voltadas à garantia da segurança alimentar na atenção primária à saúde é essencial, oferecendo espaço para debate, intervenção precoce e acompanhamento contínuo das famílias.

A priorização da atenção a indivíduos e famílias em insegurança alimentar tem ocorrido nos âmbitos do Sistema Único de Assistência Social (SUAS), do Sistema Único de Saúde (SUS) e do Sistema Nacional de Segurança Alimentar (SISAN), com a criação de instrumentos como a Triagem para Risco de Insegurança Alimentar (TRIA) e de orientações específicas na política de assistência social ^29^. Tais medidas fortalecem a articulação entre os sistemas, favorecendo respostas intersetoriais mais eficazes que integrem o cuidado em saúde, a proteção social e o acesso a alimentos adequados e saudáveis, contribuindo para a melhoria das condições de vida e para a redução das desigualdades que afetam essas famílias.

Entre as possíveis limitações deste estudo, cabe considerar que a insegurança alimentar reflete experiências dos últimos três meses no acesso a alimentos pelas famílias, enquanto os dados nutricionais, como peso e, especialmente, estatura, se acumulam ao longo de períodos mais prolongados. Para minimizar esse efeito, foram utilizadas medidas autorreferidas de peso e estatura, assegurando maior comparabilidade entre os dados. Ainda, as análises consideraram categorias amplas de IMC. A representatividade territorial dos dados baseia-se nas regiões administrativas, e não em bairros, o que pode restringir análises mais granulares sobre desigualdades intraurbanas ^13^. Assim, os resultados refletem padrões gerais da população estudada, mas podem não capturar todas as nuances locais ou mudanças recentes nas condições alimentares das famílias.

## Conclusão

As mulheres em insegurança alimentar apresentaram mais que o dobro do risco de apresentar obesidade enquanto em homens essa relação não foi identificada. Esse resultado sugere uma maior vulnerabilidade das mulheres aos efeitos da insegurança alimentar no contexto urbano, refletindo desigualdades de gênero que permeiam as condições de vida e saúde no Município do Rio de Janeiro. Investigações futuras são necessárias para aprofundar a compreensão dos determinantes sociais e metabólicos que diferenciam a experiência da insegurança alimentar entre os gêneros. Tais estudos são fundamentais para elucidar as causas subjacentes da obesidade em distintos grupos populacionais.

Destaca-se, ainda, a necessidade de abordagens interseccionais e multiprofissionais no cuidado às pessoas com excesso de peso. Os profissionais devem ser capazes de promover práticas e ambientes alimentares adequadas e saudáveis, considerando as desigualdades de gênero, raça e classe que atravessam os contextos de vida das famílias, em especial aquelas chefiadas por mulheres, que frequentemente enfrentam obstáculos adicionais nessa relação paradoxal entre a insegurança alimentar e obesidade.

## Data Availability

Os dados de pesquisa não estão disponíveis.
